# Lessons Learned from the 2019 Nebraska Floods: Implications for Emergency Management, Mass Care, and Food Security

**DOI:** 10.3390/ijerph191811345

**Published:** 2022-09-09

**Authors:** Eric E. Calloway, Nadine B. Nugent, Katie L. Stern, Ashley Mueller, Amy L. Yaroch

**Affiliations:** 1 Gretchen Swanson Center for Nutrition, Omaha, NE 68154, USA; 2 Nebraska Extension, University of Nebraska-Lincoln, Lincoln, NE 68588, USA

**Keywords:** mass care, emergency support function 6, food and water, flooding, emergency management, food security, lessons learned

## Abstract

This qualitative study aimed to understand the actions, challenges, and lessons learned for addressing the food and water needs of flood survivors, with a special focus on vulnerable populations and the implications for food security, to inform future disaster response efforts in the U.S. Semi-structured in-depth interviews were conducted from January to August 2020 with the local, state, and national stakeholders (*n* = 27) involved in the disaster response to the 2019 Nebraska floods, particularly those involved in providing mass care, such as food, water, and shelter, for the flood survivors. The challenge themes were related to limited risk awareness and apathy, the large scope of the impact, the difficulty with coordination and communication, the challenges in risk communication, the limited local-level capacity, and the perceived stigma and fear limiting the utilization of governmental assistance. The mitigation recommendations included the need to consider zoning and infrastructure updates, the implementation of efficient systems that leverage technology for coordination and communication, and guidance on how to address certain human factors. This study reinforces previous findings related to flood disasters and adds to our understanding of disaster response and food insecurity. The practical takeaways from this study can inform future flood-related disaster mitigation approaches in Nebraska and other rural areas.

## 1. Introduction

### 1.1. Introduction to Flooding Disasters and Response

Floods are a leading cause of property damage and death due to disasters, and they disproportionally affect populations with low socioeconomic status [1,2,3]. In recent years (2007–2019), flooding in the United States (U.S.) has caused the deaths of over 1200 people and USD 100 billion in damage [4]. Furthermore, recent trends indicate an increasing flooding frequency in the eastern Great Plains and Midwest regions of the U.S. [5,6,7,8], and these trends are expected to continue into the upcoming decades [9]. In March 2019, winter storm “Ulmer” spread over Nebraska (and the rest of the eastern Great Plains and Midwest regions of the U.S.) and contributed to one of the worst flooding disasters in the state’s history. Flood-impacted households faced displacement, food insecurity, and financial hardships that will likely take years to recover from, if at all. It is important to understand how best to prepare for, and respond to, these disasters to mitigate flooding impacts on households in affected areas.

Disaster response can be characterized by the four phases of emergency management—preparedness, response, recovery, and mitigation [10]. *Preparedness* involves activities related to pre-planning and resource allocation. *Response* involves activities that occur during a disaster, such as addressing immediate needs, saving lives, and minimizing damage. *Recovery* involves long-term activities aimed at returning affected areas to pre-disaster conditions. *Mitigation* involves activities following a disaster that focus on reducing future risk. The timing of these phases can vary across disasters, can overlap within disasters, and can sequence differently across communities and population groups affected by the same disaster [11,12,13]. Furthermore, phases such as recovery and mitigation naturally overlap, and recovery itself can be composed of activities to address short-term stability and activities to promote the long-term return to the pre-disaster state [12]. Despite the difficulties in assigning clear distinctions between these phases, to provide some guidance for the reader the phases in this study are delineated in Table 1, which outlines key events in the 2019 Nebraska floods at the time of the study. Preparedness occurs up to the point that the statewide emergency was declared; response occurs during the period of heavy flooding throughout most of the state (13–23 March); and the recovery and mitigation efforts begin after the flood waters initially begin to recede in mid- to late March.

In the case of catastrophic flooding, such as the 2019 Nebraska floods, disaster response activities must be coordinated across a large geographic area and among groups that have varying levels of expertise, resources, or history of working together. Responding entities may include governmental agencies, non-profit organizations, businesses, faith-based groups, and volunteers that may operate at the local, state, and national levels.

Disaster response activities can be categorized using the Federal Emergency Management Agency’s (FEMA’s) National Response Framework, which outlines seven *community lifelines* to be prioritized for stabilization efforts [14]. Particularly relevant in the context of flooding impacts on populations with low socioeconomic status is the *mass care*, *emergency assistance*, *temporary housing*, *and human services* lifeline, referred to as Emergency Support Function (ESF) 6 [15]. Specifically, ESF 6 involves activities, such as congregate sheltering and feeding; distribution of emergency supplies; coordination and management of volunteers and donations; rehousing; administering disaster assistance programs (e.g., governmental nutrition assistance, disaster unemployment benefits, funding for repairs, etc.); and other related functions. These activities, and the planning and coordination of them, occur throughout the four phases of emergency management. Henceforth, when the term “disaster response” is used, it refers to activities that fall within ESF 6 and may refer to any of the four phases of emergency management (not just “Response”).

While the 2019 Nebraska floods took a heavy emotional and financial toll on affected households, they also offer an opportunity to learn lessons about disaster response efforts. Low socioeconomic status populations may be relatively more vulnerable and in need of ESF 6 activities due to being less able than other groups to acquire alternative sources of food and water and recover financially [16,17,18,19]. Existing issues related to food and water security may be exacerbated by flooding disasters. Providing effective mass care during flooding disasters and recovery can reduce the long-term impacts of disasters on vulnerable households. While the relationships between disaster recovery, mass care, and food security have been investigated internationally and in developing countries, there has been limited research investigating mass care in the context of food insecurity in the U.S. Therefore, this study aimed to understand the actions, challenges, and lessons learned for addressing the food and water needs of the flood survivors, with a special focus on vulnerable populations and the implications for food security, to inform future disaster response efforts in the U.S.

### 1.2. Background on the 2019 Floods in Nebraska

The March 2019 bomb cyclone, a rapidly intensifying low-pressure weather system referred to as winter storm Ulmer, occurred during the wettest 12-month period in U.S. history. Prior to Ulmer, in late November 2018, the Nebraska Emergency Management Agency (NEMA) predicted problems with ice jams on rivers throughout the state. By March 2019, 90% of Nebraska’s rivers were jammed with ice and above-average flooding was predicted as the weather warmed and the ice melted. Seasonal predictions did not, however, account for the massive low-pressure system that followed on March 12th and 13th, leading to a rapid thaw of ice and snow, hurricane winds, and blizzard conditions across the state.

The subsequent floods led to the deaths of five community members and over USD 3 billion in damages to infrastructure (e.g., roads, bridges), commercial and personal property, and agriculture (e.g., livestock, crops, grain, and feed). As a result, there were state or federal disaster declarations in 104 cities, 81 (of 93) counties, and five tribal nations. Additionally, the emotional and financial toll on Nebraska’s communities was considerable, as many households suddenly found themselves jobless, homeless, and/or facing immense financial hardships.

## 2. Materials and Methods

### 2.1. Overview

We conducted 26 semi-structured in-depth interviews (and one additional open-ended text response from a stakeholder who did not want to be interviewed) from January to August 2020 with stakeholders who were involved in various aspects of the disaster response to the 2019 Nebraska floods. The qualitative approach falls within phenomenological research [20]. We sought to understand the lived experiences of those that responded to the flooding disaster, including the actions taken, the challenges encountered, and the lessons learned as they related to providing food and water to those impacted by the floods. Study activities were conducted in accordance with the prevailing ethical standards, and the study was deemed exempt from review by the University of Nebraska Medical Center Institutional Review Board for Human Subjects Research.

### 2.2. Sampling and Perspective

The interviewees were involved in activities related to ESF 6 (e.g., food, water, and shelter needs). First, we conducted two unrecorded formative interviews with individuals with first-hand and comprehensive knowledge of the overall disaster response in Nebraska to understand the pertinent events and information related to the disaster response. We supplemented these formative interviews with documentation from news stories and relevant websites. We also used this formative work to pilot an initial interview guide and to inform the initial list of candidate interviewees. The two formative interviewees were not included in the analytic sample but did participate in member checking. The subsequent interviewees were identified via snowball sampling.

A total of 27 of the 42 invited individuals provided data for the final sample. The sample consisted of those with a *local-level perspective* (emergency managers (*n* = 3), elected local government representatives (*n* = 2), leaders of non-governmental community organizations (*n* = 4), non-governmental community volunteers involved in leading local response efforts (*n* = 3), and leaders of long-term recovery groups (*n* = 4)); *state-level perspective* (governmental agency representatives (*n* = 3) and leaders of non-governmental organizations (*n* = 3)); and *national-level perspective* (leaders of non-governmental organizations (*n* = 4) and one representative from a large grocery retailer who led the response efforts of their company). All local-level interviewees lived and/or worked in counties impacted by the floods.

### 2.3. Procedure

We conducted the semi-structured interviews by telephone; they were audio recorded and approximately 60 min in length, and we transcribed them verbatim. One participant, who was uncomfortable being interviewed, instead submitted written responses to the questions. Three of the study authors conducted the interviews (one author per interview). Table 2 lists the primary questions covered in the interviews. Based on each interviewee’s contextual experiences in the disaster response, the interviewers utilized probing follow-up questions (e.g., “tell me more about why that was important.”) to elicit rich responses.

The qualitative approach incorporated thematic analysis utilizing Creswell’s “lean coding” technique [21]. The only a priori framework utilized in the analysis was framing in line with the four main phases of emergency management (i.e., *preparedness*, *response*, *recovery*, and *mitigation*). Within those four categories, themes were allowed to emerge inductively. The study authors met regularly to discuss preliminary interview findings and noted emerging themes, meanings, and relationships among the themes. Once no new major themes emerged, the authors determined that theoretical saturation was reached.

Once all the interviews were completed, formal qualitative coding and analysis proceeded in three iterative steps. First, the three interviewers read and open-coded two randomly selected interviews. The interviewers then met and used open-coding memos to draft an initial coding list. Operational definitions and examples were discussed for each initial code. The initial coding list was used to code two randomly selected interviews. Second, the interviewers met to discuss the adequacy of the coding list—removing, moving, combining, redefining, and adding codes where needed. The interviewers then each coded another randomly selected interview and again met to discuss the adequacy of the code list and definitions. Third, with only minor modifications needed, the same three interviewers coded the remainder of the transcripts and written text responses using the full codebook.

In the analysis, the emergent themes were described, along with the illustrative quotations. The theme descriptions were organized by the four phases of emergency management and around the key challenges faced for preparedness, response, and recovery. For mitigation, the themes were related to interviewee recommendations and lessons learned. Due to funding and timeline limitations, member checking was limited in scope. An initial draft of the findings was presented to one of the formative interviewees who provided a member check. This formative interviewee agreed with the findings and provided input on clarity and terminology use.

## 3. Results

The following sections describe the themes related to preparedness, response, recovery, and mitigation. The interviewees described primary activities performed, challenges encountered, and recommendations for mitigation based on their experience with the 2019 Nebraska floods. See Table 3 for an outline of the emergent themes.

### 3.1. Overview of Disaster Response, Emergency Management Activities

#### 3.1.1. Preparedness Activities

The interviewees reported that primary disaster preparation activities involved pre-planning, pre-communication, and/or training at the organizational, local, and state levels. The planning ideally included multi-sector planning committees to talk through disaster scenarios and responses. The local-level activities were typically led by the county emergency manager. The state-level activities were primarily carried out by state agencies and the Nebraska chapter of the National Voluntary Organizations Active in Disasters (NE VOAD).

Another preparation activity was the monitoring of weather trends. The interviewees who worked for disaster response organizations used National Weather Service data to monitor conditions and collect primary data using on-the-ground assessments (e.g., from local emergency managers), river gauges, and drone footage. Based on the available weather information, past trends (e.g., data from the past 3–5 years) were used to identify potential high-risk areas. The weather information was used to warn emergency managers and others in high-risk areas, to inform the strategic placement of resources (e.g., trailers, emergency response vehicles, food, other mass care supplies, etc.) and disaster response staff/volunteers, and to coordinate with organizations/agencies to address anticipated needs.

#### 3.1.2. Response Activities

According to the interviewees, disaster response varied by locale, and several interviewees described local leaders forming informal committees to quickly plan and execute disaster response activities. At the state level, the Nebraska Emergency Management Agency (NEMA) worked to coordinate with those on the ground, such as emergency managers and other leaders, to identify mass care needs. Additionally, various disaster response organizations and social service agencies worked to procure and organize food and water donations.

Food banks, restaurants, corporate manufacturers and retailers, and individuals provided food and water donations. Contributions were procured locally as well as from across the state and country. In many impacted towns, hubs for donations were set up at fire stations, schools, or other locations central to the communities. Community leaders relied on various means of communication (e.g., local news/newspaper, radio, telephone trees, social media, school email listservs) to inform community members about available resources and to request additional donations.

Congregate meals were provided to individuals staying in shelters, volunteers who were assisting with recovery efforts, and other impacted community members. These meals were formally convened through disaster relief organizations at shelters or as part of donations from local restaurants, religious congregations, or community members who had the means to help. Additionally, unprepared and shelf-stable food donations were provided at community hubs, local food pantries, and via mobile pantries.

#### 3.1.3. Recovery Activities

Several multi-agency resource centers were established throughout affected areas in schools, community centers, warehouses, libraries, places of worship, and even vacant commercial buildings (e.g., “an old Sears store”). The multi-agency resource centers served as resource hubs for local organizations (e.g., faith-based, hunger-relief, community-based organizations) and federal agencies or national organizations (e.g., Department of Health and Human Services (DHHS), Salvation Army, Red Cross, United Way, and FEMA) to provide a “one stop shop” for affected households. Approximately, 30–50 organizations/agencies were represented at the multi-agency resource centers, and they provided services such as case management, financial assistance, housing referrals, document replacement, and registration for the Disaster Supplemental Nutrition Assistance Program.

The recovery process also included the establishment (as per guidance from FEMA) of a 501c3 non-profit organization within each county dedicated to long-term recovery. These were referred to as long-term recovery groups, and they served as receiving hubs for donated funds and other resources. The long-term recovery groups coordinated the direct delivery of donations to people in need and established temporary distribution centers to house donations.

### 3.2. Disaster Response Challenges

#### 3.2.1. Preparedness Challenges

*General apathy.* The primary challenge the interviewees discussed for preparation activities was general “apathy” towards participating in pre-planning and pre-communication activities. This was primarily discussed in the context of local-level activities and to a smaller degree within organizational planning. The interviewees reported a lack of understanding among some local-level community leaders of the importance of disaster planning and how they could get involved or contribute in a meaningful way. One interviewee described their attempts to hold regular community disaster planning meetings in their county, “I was trying to… get the buy in from the people that are supposed to be involved. It didn’t happen… I would only have two people show up, and it’s hard to call it a committee when it’s just three people there.” The interviewees attributed this apathy to three main causes: the long duration since the last major disaster, the limited capacity, especially in smaller communities (where local leaders may “wear many hats”), and personnel turnover contributing to loss of experiential disaster knowledge.

#### 3.2.2. Response Challenges

*Large scope of impact.* A central challenge posed to the response efforts was the overall scope and impact of the flooding event. At the local level, response efforts were sometimes stymied due to inadequate preparation. As one interviewee stated, “Our community was not a flood community that had participated and done things that were supposed to be done in the flood plain because we didn’t have to. It’s just the way it was.” In other cases, a disaster plan did exist, but it did not include sufficient flooding considerations. One interviewee explained this as, “… we haven’t had a disaster for so long. Usually our disasters are tornadoes, and we really didn’t know who was in charge of [flood response] and what we needed to do and where.”.

*Damage to infrastructure causing isolation.* The damage the flood left in its wake also affected the immediate food and water response. The damage to infrastructure, such as roads and bridges, made it difficult for disaster relief organizations and donations to reach impacted areas. In the case of Fremont, the flood waters surrounded the city and damaged exit routes, which left the city isolated for a short period. Many communities had to “fend for themselves” during the initial several days of the floods as disaster relief organizations faced difficulty reaching communities and/or faced delays in scaling relief efforts to the scope of the need. This was especially true for many smaller and more rural communities that felt forgotten in the response efforts. As one interviewee described, “There was a lot of attention to Omaha and Lincoln and communities up and down the Missouri river. But once you got up this way, it’s like a lot of things politically as well, you know, just kind of forgotten.”.

*Difficulty managing donations.* Leaders had to convene and prioritize actions quickly (e.g., setting up shelters, resource hubs, storage for donations, etc.). One barrier mentioned by interviewees included a lack of existing relationships and proper communication with agencies facilitating donations. One interviewee mentioned the difficulty in juggling different kinds of donations, such as how to safely store both food and cleaning supplies (e.g., bleach). Additionally, while donations were appreciated, the interviewees spoke of the difficulty in fielding the large volume of donations, the excesses of certain items (e.g., bottled water), and/or inappropriate donations such as expired foods, less healthful foods (e.g., junk foods), and used furniture.

*Language barriers and immigration concerns.* Language barriers also presented a challenge. Many towns had large populations of non-English speaking residents. The interviewees reported that these populations were not as informed about the actions they should take and the resources available due to a lack of language-specific outreach. Moreover, in some areas it was reported that addressing language barriers had not been adequately anticipated by some national disaster response groups. Local organizations that worked with non-English speaking groups were called on to fill the gap of available interpreters. Further, the interviewees expressed that some community members were afraid to seek assistance due to fears about “public charge” and deportation risk.

#### 3.2.3. Recovery Challenges

*Difficulty establishing long-term recovery groups and maintaining recovery momentum.* One key challenge reported by the interviewees during recovery was the difficulty in establishing county-level long-term recovery groups. The interviewees reported struggling to establish these non-profits due to limited experience and/or capacity. One long-term recovery specialist described forming the 501c3, “Talk about a pain… we had to get articles of incorporation… an IRS number… It’s been hard.” Multiple 501c3s may have reportedly diluted overall recovery assistance and created an unintended competition for resources among affected counties across the state. Smaller communities with less infrastructure or support systems fell short during recovery. Sustaining incoming donations and recovery efforts was difficult as public interest began to wane, media coverage subsided, and volunteers experienced burn out. One interviewee explained, “This was really eye opening to me about flood relief is three to four weeks in, it isn’t really a thing to a lot of people anymore. These people are in need right now and, ‘Oh well now the water is subsiding, so the need has gone away.’ And that is actually the very opposite.” Lastly, the long-term recovery efforts were interrupted by a new public health emergency, in early 2020, approximately one year post-flood, with the emergence of the COVID-19 pandemic. One long-term recovery group stakeholder explained the impact on long-term recovery, “And then in this case, it’s impacted [because of] COVID-19 for the simple fact that that is the big issue right now. Flooding is not important anymore. It’s COVID-19.”.

*Stigma, fear, and limited awareness impacting assistance utilization.* Underutilization of assistance due to perceived stigma, fear, and lack of public awareness was also a challenge. Interviewees reported that there was stigma, among community members, associated with asking for food assistance (e.g., Disaster Supplemental Nutrition Assistance Program benefits, food pantry donations, etc.). This could be especially prevalent in rural communities and small “close knit” towns where it can be more difficult to keep this information confidential. The interviewees also explained that not asking for food assistance often meant that families went without or relied on less expensive and often less healthful choices. One recovery specialist described, “Especially in the rural areas and probably the whole state, people are super proud. And to ask for help or to get help, that’s very hard for people. Most of these are first timers, so they’ve never asked for public assistance or a donation ever in their life.” Distrust, fear, and frustration with governmental assistance was also common among the recent immigrants from the Latino communities, which can be large and undercounted in many rural areas. Noted concerns included fear of potential deportation, worry that using governmental assistance would impact their path to citizenship, and frustration due to language barriers. The interviewees also stated that governmental (e.g., Disaster Supplemental Nutrition Assistance Program) food assistance and non-governmental (e.g., food pantries) food assistance were underutilized because the available aid was not communicated broadly (e.g., some people did not use social media or listen to the radio). For example, in one county, “The issue [was] not lack of food or lack of water. It was making sure that the families who needed it most were aware of it.” Other households did not understand the application process to receive Disaster Supplemental Nutrition Assistance Program benefits or assumed that they would be ineligible. “Middle class families are being impacted with loss of jobs and they’re needing help with food security and energy assistance. And they don’t know how to use the system. They don’t know about [Special Supplemental Nutrition Program for Women, Infants, and Children (WIC)]. They don’t know about [Supplemental Nutrition Assistance Program (SNAP)].”.

*Administrative barriers to accessing federal recovery funds*. The interviewees described having difficulty securing federal funds for recovery efforts at the household and community level. A common perception of the interviewees was that federal disaster relief organizations provided inadequate or uncoordinated support, specifically among economically vulnerable residents. Many households did not qualify for financial assistance because, they were told, the damage was not severe enough. Moreover, many families who rented property did not have flood insurance (which was reportedly very expensive) and/or were ineligible for other reasons (e.g., undocumented residents). In several instances, individuals ineligible for financial assistance from FEMA incorrectly assumed that they were also ineligible for assistance from other organizations. One long-term recovery specialist explained, “[We had many] families who were told they couldn’t receive any FEMA [assistance] and were unaware of all the other services that were available. I think better education [is needed] through the Multi Agency Resource Center system to say, ‘Hey, just because one organization says they don’t help, there’s other support systems that we can help you with.’” Perceived long timeframes for processing requests delayed the securing of federal recovery funds. One interviewee described this as, “It’s so frustrating. …they say they’re not going to get the money until next summer, it may be December 2021, these people can’t wait that long.’ …there’s a huge disconnect, there’s all this money, but it’s not fast enough and it’s not flexible enough.”.

### 3.3. Mitigation Recommendations

#### 3.3.1. Prepare for Large-Impact, Low-Frequency Disasters

The interviewees expressed the need to “think big” during planning and preparation activities. The available historical data and experience were not sufficient to inform preparation for a disaster of this scale. An interviewee advised the disaster response field to “…think big all the time,” and then went on to say, “… [we didn’t think big] because we relied just on our data and previous experiences.” The interviewees stressed the importance of planning for the storing and distributing of food, water, and sheltering equipment on a large scale. In addition to planning, the interviewees advised on the importance of investing in modernizing the infrastructure and equipment related to transportation, drainage, city water, communications, and rescue to be able to handle large-scale disasters. An interviewee recommended reminding stakeholders that when disasters happen, everyone ends up paying for repairs, and so, it is best to invest in infrastructure that will last, “Whether you had stuff in the floodplain or not, you’re still paying for it [in taxes]. Look at the amount of damage for roads and bridges that were not in compliance and got wiped out, we’ve got to build them right, so we don’t have to build them back again.” Moreover, coupled with infrastructure improvements, the interviewees advised that local zoning could be an important tool to ensure that development is conducted in lower-risk areas.

#### 3.3.2. Enhance Local-Level Capacity to Plan, Respond, and Recover

Many communities found themselves initially isolated from outside assistance. This varied by community and reportedly lasted anywhere from several days to a week or even the whole duration of the response and recovery efforts. Moreover, the interviewees perceived disparities across communities in disaster response and resource allocation. Therefore, the interviewees recommended building local-level capacity to prepare, respond, and recover from disasters, especially for large-scale events when the response will likely be stretched thin. One interviewee urged communities to establish task forces to collaborate on plans, “[Communities need] to develop a disaster task force that knows the process. Where are we putting food and water? Where is the first shelter until Red Cross can get here? Who’s in charge of it? … [This way] we already know what we need to do.” In line with the “task force” suggestion (but perhaps more permanent), several interviewees suggested that all communities establish COADs (i.e., Community Organizations Active in Disasters) made up of emergency manager(s), local organizations, agencies, businesses, and community leaders. The COAD would develop local-level disaster planning and could transition into a long-term recovery group following a major disaster. One interviewee urged communities to “…stand up COADS, well before the disaster …It is extremely difficult for these communities to figure this out after the disaster has happened.”.

#### 3.3.3. Build Inter-Organizational Relationships and Communication Channels

One of the more prevalent recommendations was to cultivate and nurture inter-organizational (and interpersonal) relationships among those responding to disasters, and to do this well before disasters took place. One interviewee described this as, “Definitely one of the most critical things is to have those relationships in advance… If we’re in the situation, can we get food from you? Can we get water from you? And if the tables are turned, we’ll do the same for you.” Several organizations reported having internal disaster response plans with who to call and what to offer (or ask for); however, other organizations were sometimes unaware of these plans. An interviewee from a state-level anti-hunger organization described an exchange with a representative from a large national organization who was unaware of the anti-hunger organization’s capabilities, “You need that connection piece [between organizations] … we were a couple months in and out in Fremont, working with [large national organization] and they were like, ‘We didn’t even know we could ask you to do this.’ That kind of thing. Well, we also have ice to offer, refrigerated tractor trailers [to store] donations, we have [commercial driver’s license] drivers that we could lend, we have volunteer management coordinators on staff… So, they didn’t even know.” Moreover, forming relationships with organizations that have a local “boots on the ground” presence (e.g., university extension offices) can reportedly assist with gaining local knowledge to “ground truth” data during disaster response. The interviewees recommended transparent and inclusive planning around organizational collaboration in disaster response. A framework to guide these relationship-building and communication efforts described by the interviewees was FEMA’s seven lifelines. At the time interviews were conducted, the interviewees reported that Nebraska was working to implement the seven lifelines approach.

#### 3.3.4. Enhance Mass-Communication Capacity among Responders and to the Public

The interviewees conveyed that efficient communication was key to effective collaboration. The interviewees recommended implementing and using technology that enables mass communication among those responding to disasters. Moreover, they indicated that a means to easily inform large numbers of the public to provide information and updates specific to their locale was needed—similar to an “Amber Alert” system. An interviewee reported that Nebraska was working on such a system at the time of the interviews, “They’re working on [a system that] first responders would have and Emergency Managers, it’s called First Alert. They would get priority and anybody else…” Many interviewees that were tasked with public outreach on various aspects of the disaster reported using telephone calls, social media sites, traditional media (e.g., radio, TV, print, etc.), and door-to-door approaches to get the word out about risks and resources. While the interviewees acknowledged these traditional modes would still be needed, they suggested approaches that leveraged technology and smart phones in public outreach activities.

#### 3.3.5. Leverage Technology to Coordinate Large-Scale Influx of Disjointed Donations

An issue reported by several interviewees, especially those working at the local level, was the seemingly uncoordinated way in which non-local donations would arrive in their communities. Donations could arrive with little forewarning and many times did not match local needs. This led to waste and surpluses of unneeded items. An interviewee said, “In hindsight it would be awesome to have some sort of coordinated effort or somebody who says ‘Okay, here’s what Kearney needs or here is what Buffalo County needs. It’s estimated they need 10,000 bottles of water, diapers, ready-to-eat meals, etcetera.’” To facilitate coordination, the interviewees recommended implementing a centralized web-based system (with accompanying physical intake and distribution centers) to handle intake, processing, ordering, fulfilling, and tracking of non-local donations. Such a system could serve as a repository for donations so that communities could request needed resources and locate available volunteers. The interviewees also noted that having a centralized donation system could streamline donor education efforts where specific needs could be indicated to those that wished to donate. At the time of the interviews, Nebraska had implemented a web-based platform called Knowledge Center as a step toward centralizing the assessment of the needs and connecting groups that could meet those needs.

#### 3.3.6. Raise Public Awareness and Individual Capacity through Inclusive Outreach

The interviewees that worked with affected community members reported that many were unaware of disaster preparedness, what they should do when flood warnings occurred, and how they could get help. An interviewee described this as, “…much of the effort that happened was reactive… [it would have been helpful] if that were something we regularly communicated about before. These are the options, this is how you qualify, and what the process is, specifically for food access… because there was a significant amount of people that had never experienced food insecurity and had no idea where to go.” The interviewees recommended implementing routine public education and awareness campaigns to inform households about the basic disaster preparation and response actions they should take. Furthermore, at timely intervals, such as when conditions indicate flooding may be likely, more intensive and targeted outreach efforts may be beneficial. For example, community forums were held in Nebraska in early 2020 when it appeared that conditions might be in place for a similar flooding disaster. Finally, the interviewees cautioned that any community outreach efforts should include a broad and inclusive approach that ensured that all members of the community received the needed information regardless of language, literacy, living situation, immigration status, disability, or other barriers to communication. The interviewees recommended using a diverse public education task force that represented the various sub-groups within communities to plan outreach efforts.

#### 3.3.7. Considerations around Setting Up Shelters and Resource Sites

The interviewees suggested setting up sites near affected areas to reduce transportation barriers. They advised non-local organizations and agencies to work with local partners to determine the best locations. Leveraging local knowledge could also help prevent outside groups from duplicating existing local efforts. Once sites are chosen, the interviewees cautioned that site organizers need to be mindful of issues such as stigma (especially in small rural towns where using governmental assistance can be taboo) and lack of familiarity with assistance programs among some affected community members and issues such as fear and language barriers. The interviewees suggested that site organizers could reduce stigma by offering a range of services (so that conceivably a person could be seeking other types of services, not only Disaster Supplemental Nutrition Assistance Program benefits, for example) and should ensure privacy for those seeking help. To address the lack of familiarity, the interviewees recommended providing trained guides at resource sites, especially multi-agency resource centers, to orient and assist affected households. Lastly, the site organizers could make recent immigrant populations feel safe and welcomed by working with local organizations that serve those populations to conduct outreach; ensuring interpreters are available and forms are in multiple languages; and being mindful that governmental uniforms and/or insignias may discourage participation. An interviewee from a local organization working with immigrant groups described their experience as, “[When conducting outreach] We had to do a lot of door-to-door almost coaxing, ‘Hey, you can’t be here. This is not safe. Here’s a shelter you can go to, and we’re going to be there, and we are going to be helping.’ People were just scared in general that Immigration [and Customs Enforcement] would come in… I would think that [external organizations coming into a] community should have a local partner that can help with cultural things and language barriers.”.

#### 3.3.8. Considerations around Providing Food and Water

The interviewees discussed the varying nature of the food needs depending on timing and living situations. Food needs were reportedly highest during the first few days, coinciding with sheltering needs. In this situation, ready-to-eat foods and shelf-stable snacks were most needed. The need extended to volunteers who helped with cleaning and rebuilding. As community members began to be re-housed, the food needs varied based on housing situations (e.g., home, spare room, “couch surfing”, hotel/motel, camper, car, tent, etc.). Food donations needed to be tailored to the household’s available food preparation and storage equipment as well as their preferences. For example, an interviewee conveyed that some households lived out of campers for over a year after the initial floods. These households relied on propane as the sole cooking source, which can be cost-prohibitive. This interviewee explained, “A lot of the donations are rice. And people tell me they don’t want the rice because it takes so long to cook, and it’ll cost them $20 for free rice.” The interviewees suggested that once community members were out of the shelter, an ideal approach to tailor donations to meet specific needs was through cash vouchers/gift cards to a nearby grocery store. Finally, the interviewees warned that food pantries in affected areas should prepare for increased traffic starting a few months after the disaster to a year or more as the financial ramifications of the floods took their toll on affected households.

The water needs varied by community. Many communities did not see much need for bottled water after people vacated shelters and/or after clean-up volunteers had finished immediate response efforts. Excessive bottled water donations were reported by several interviewees, and some were still trying to get rid of the excess water over a year later. In other communities, city water systems were damaged, and community members in these areas relied on bottled water for hydration and hygiene needs. Another approach was to use food-grade water tanker trucks to refill city water supplies. There were mixed results reported due to contamination levels or sand accumulation in water pipes. The interviewees suggested that water donations should be coordinated and targeted to areas in need.

## 4. Discussion

The focus of this case study was to qualitatively explore the 2019 Nebraska floods’ disaster response to understand the actions, challenges, and lessons learned for addressing the food and water needs of the flood survivors, with a special focus on vulnerable populations and the implications for food security, to inform future disaster response efforts in the U.S. Key challenges were related to the limited risk awareness and apathy inhibiting preparation activities, the large scope of the impact and infrastructure damage, the difficulty with coordination and communication among the responding agencies and organizations, the challenges in risk communication, the limited local-level experience and capacity, and the perceived stigma and fear limiting the utilization of assistance. The mitigation recommendations largely stemmed from these challenges and included the need to consider zoning and infrastructure updates, to implement efficient systems that leverage technology where possible for coordination and communication, and to provide guidance on how to address certain human factors during disaster response.

Early planning and preparedness exercises can be crucial to response and recovery effectiveness [22,23,24,25]. The interviewees reported low risk awareness and limited bandwidth in communities and among organizations and agencies as primary barriers to local-level preparedness. Limited community and organizational planning have been reported elsewhere in communities in the midwestern U.S. [26] and a large urban U.S. community [27]. With increased awareness of flood risk in Nebraska, following these floods, this may be an opportune time to support local-level disaster preparation activities and to establish new social norms around preparation.

The interviewees in the current study emphasized the need to prepare for large-scale catastrophes. Cases studies of the 2010–2011 flooding in the state of Queensland, Australia, described another widespread flooding disaster across a rural area that damaged infrastructure, isolated communities, and impacted food [28,29]. Similar outcomes occurred in both floods, including the emergence of informal local-level food assistance and recovery volunteerism, forced community self-reliance due to isolation, and the need for massive cross-sectorial and multi-level emergency response [28,29]. Key lessons learned and/or barriers during the Queensland floods were the need for multiplicity in transportation infrastructure, the inherent vulnerabilities of long food supply chains with “just in time” stocking, the inadequacy of communication systems among responding entities, and the need to incorporate multi-stakeholder collaboration in the disaster preparation and mitigation efforts. Nebraska food systems were tested to a similar extent, and the lessons learned hold largely true in both contexts; however, the food systems in Nebraska were less severely impacted compared to those in Queensland. In a study of an urban environment, similar factors were described as important for food system resilience in response to disaster, such as planning, training, backup infrastructure, communication systems, inter-organizational relationships, monitoring systems, and post-event assessment and application of lessons learned [27].

While the food systems in Nebraska proved to be somewhat more resilient than those described in the Queensland case studies, the interviewees in the current study emphasized similar limited coordination between local-level (formal and informal) entities and non-local entities. This was most evident in issues coordinating non-local food and other donations and the duplication of local efforts by non-local groups. While local entities, especially in smaller municipalities, may have limited staffing, rely on voluntary officials, and have fewer financial resources [30], they also have the advantages of more nimble processes and local knowledge [28]. For large-scale disasters that increase the likelihood of community isolation, building the local capacity for maintaining food security may be crucial. Multiple and varied sources of food, wider partnerships across local and non-local entities, better connected organizations, and more efficient and effective flows of information may contribute to better responses to food insecurity [31].

The time period that follows a disaster offers an opportunity to reinvigorate disaster preparedness and mitigation efforts [32,33]. Some researchers estimate that societal salience of disaster events provides a 2- to 8-year window to pursue changes in policy, practices, and/or infrastructure [32,34], and this window may be relatively longer for large-scale disasters [26,35]. State agencies can be crucial in passing policies to enforce mitigation actions and in connecting local entities [22,36], but most flood mitigation activities occur at local levels [30,36]. Moreover, while state policies can preempt local policies in many instances, flexible, rather than prescriptive, policies can allow communities to tailor solutions to their local needs [22,36]. The ability to assess the lessons learned from disasters, conduct in-depth and deliberate processes to identify mitigation strategies, and approach issues from a system-wide perspective may lead to more resilient communities in the long-term [26,37,38]. Inter-community, inter-agency, and public engagement in these processes can be crucial to promoting effective policy change, mitigation action, and allocation of resources to support activities [26,28,36,39,40].

It has been reported that smaller and rural communities, such as the majority represented in this study, tend to prefer structural mitigation and non-structural emergency response approaches [30,41]. In this study, the interviewees proposed structural and non-structural mitigation approaches. Structural mitigation, such as levees, can have positive and negative outcomes. While they can offer an efficient means to reduce flood risk in certain areas, they can have unintended consequences, such as lowering risk perception and disaster preparation participation among the public [32,42]. The installation of levees, in particular, can exacerbate higher water levels, leading to less common but more catastrophic floods [43], and can increase the flood risk for down-river communities who are not protected by them [26]. The interviewees also proposed non-structural mitigation approaches, which included public awareness and education campaigns, zoning policies to limit building in flood plains, enhanced coordination among responding organizations, and improved communication systems. Additional non-structural approaches may include wetlands restoration, supporting riparian buffers, and other natural land and river management approaches [44,45,46]. A combination of structural and non-structural approaches may offer complementary solutions and prove to be a promising mitigation approach [44].

Financial resource constraints can limit mitigation efforts, particularly in rural communities. Rural areas in the U.S. tend to have less affluent populations on average, a smaller tax base, and weaker relationships with state and federal governmental entities [19,47] and therefore may need to seek financial assistance to fund structural mitigation efforts. For example, FEMA, through several different programs, may partially fund flood mitigation projects. However, rural areas can face barriers such as difficulty reaching qualifying damage thresholds due to low population density, the categorization of farm and ranch land as businesses, and complexities in applying for funding [26,30]. The interviewees in this study also voiced barriers to accessing federal recovery funds, but they articulated that pursuing these funds was necessary due to the widespread damage.

While preparedness and recovery-focused public awareness and education campaigns were discussed by the interviewees as non-structural mitigation approaches, there were several relevant challenges that surfaced about these efforts. The challenges included language barriers, immigration status complications, and the stigma around using government assistance programs among the target audiences. Many households do not take sufficient disaster preparedness actions, or they lack knowledge about which actions to take, due to a number of reasons [48,49,50,51,52]. Without risk communication, household preparedness decreases over time [53]. Households who do not speak English in the U.S. can be especially at risk because risk communications are often presented exclusively in English [17,54,55]. For promoting recovery resources (e.g., governmental assistance programs), survivors’ residency statuses and the stigma surrounding these resources may limit use. Households may be unable to access assistance programs and/or be hesitant to interact with federal agency officials due to immigration concerns [52]. For Latino immigrant groups in the U.S. specifically, “promotoras de salud” (i.e., community health workers), local governmental agencies, school representatives, and non-governmental organizations may be seen as trusted for outreach efforts [17,50]. In rural areas of the U.S. especially, the perceived stigma associated with receiving governmental assistance and the preference for self-reliance have been shown to impact food assistance utilization [56,57]. Tailored approaches are needed to consider these barriers to promote household-level risk reduction activities and resource utilization. Furthermore, such campaigns may be more effective when integrated alongside other policies, practices, and ongoing mitigation projects [24].

## 5. Conclusions

This case study explored the activities, challenges, and recommendations of those who responded to the 2019 Nebraska floods. This study reinforces previous findings, and it adds to our understanding of providing food and water and addressing food security issues in disaster response in the U.S. The practical takeaways from this study can inform future flood-related disaster mitigation approaches in Nebraska and other regions. Furthermore, many of the recommendations on disaster response activities gleaned from the interviewee experiences may be relevant to other types of disasters and public health emergencies.

## Figures and Tables

**Table 1:** Timeline of key events that occurred during the 2019 Nebraska floods.

Phrase	Dates	Event
Preparedness	July 2018–June 2019	Nebraska experiences the most precipitation on state record in a 12-month period.
	Late November 2018	Nebraska Emergency Management Agency (NEMA) predicts problems with ice jams on Nebraska’s waterways.
	January–March 2019	Excessive cold weather and record snowfall accumulates across Nebraska.
	11 March 2019	Winter storm Ulmer is named; moves eastward from Colorado.
	12 March 2019	Nebraska Governor Pete Ricketts declares a statewide emergency.
Response	12–14 March 2019	A bomb cyclone, a massive low-pressure system with blizzard conditions, torrential rain, and flooding, engulfs Nebraska.
	13–23 March 2019	Historic flooding impacts most of Nebraska. Initial flood waters arrive and recede at varying times across communities. Immediate response activities occur, including establishing community shelters, conducting grocery store inventory/shelf assessments, and delivering and receiving donations.
	14 March 2019	Several roads and bridges are washed away; 41 levees are breached with 350 miles of damage; Spencer Dam collapses.
	15 March 2019	Offutt Air Force Base floods, sustaining over USD 600 million in damage.
	15 March 2019	Twenty-five communities across Nebraska have full or partial evacuations.
	20 March 2019	National Guard units airdrop and truck in hay to sustain cattle during the critical calving season.
	21 March 2019	Federal disaster declaration is made (DR-4420-NE).
Recovery and Mitigation	23 March 2019	Several multi-agency resource centers open across the state during the time period from March 23rd through late April.
	19 April 2019	Disaster recovery centers (DRCs) open across Nebraska for affected residents to apply for FEMA assistance.
	8 May 2019	Crop damage and standing floodwaters stall planting season; 420,000 acres remain unplanted.
	8 July 2019	Heavy rains bring another round of major floods, resulting in property damage and evacuations for hundreds of central and southern Nebraska residents.
	20 January 2020	First recorded coronavirus disease 2019 (COVID-19) case in the United States.
	1 August 2020	Hundreds of community members still reside in temporary housing.

**Table 2:** Primary questions covered during the semi-structured interviews categorized by preparedness, response, and recovery sections, with mitigation recommendations solicited throughout the three sections.

**Preparedness**	A. What was your/your organization’s role in planning and preparation for food and water relief efforts to prepare for disasters?
	B. What were some barriers that affected planning and preparation for food and water?
	C. What was especially helpful to planning and preparation for food and water?
	D. Based on your experiences with the 2019 Nebraska floods, what advice would you give your counterparts in other states related to preparing and planning for food and water relief during disasters?
**Response**	E. What was your/your organization’s role in responding to food and water needs during the flooding?
	F. How are food and water shipments processed and distributed?
	G. How was food and water provided to hard-to-reach groups?
	H. What were some barriers that affected the food and water relief efforts?
	I. What was especially helpful to the food and water relief efforts?
	J. What are some considerations regarding the type of food (and water) that was distributed in mass care relief?
	K. Based on your experiences, what advice would you give your counterparts in other states related to food and water relief during floods?
**Recovery**	L. What kind of work are you still doing to assist those affected by flooding?
	M. Would you describe how people were impacted long-term by the flooding in terms of their ability to provide food and water for themselves/their household?
	N. Could you describe differences in how various groups have coped with the flooding?
	O. Specifically related to food security and water provision, what factors are most important for long-term recovery for community members?
	P. What were some barriers that are affecting the long-term recovery efforts related to food security and water provision?
	Q. What has been especially helpful to the long-term recovery efforts?
	R. Based on your experiences with the 2019 Nebraska floods, what advice would you give your counterparts in other states related to long-term recovery efforts?

**Table 3:** Emergent themes related to activities, challenges, and/or recommendations from each phase of the emergency management response.

Phase and Theme Type	Section in Text	Theme
Preparedness Activities	3.1.1.	Pre-planned, pre-communicated, and/or trained for disaster response.
		Monitored weather trends.
Response Activities	3.1.2.	State level: the Nebraska Emergency Management Agency (NEMA) worked to coordinate disaster response.
		Local-level: informal disaster response committees assembled ad hoc.
		Organizations and social service agencies worked to procure and organize food and water donations.
		Food banks, restaurants, corporate manufacturers and retailers, and individuals provided food and water donations.
		Donations were collected and stored at community sites.
		Community leaders informed community members about available resources and requested additional donations.
		Congregate meals were provided to those impacted by the floods and to the volunteers assisting with recovery efforts.
		Unprepared and shelf-stable food donations were provided at community hubs and local food pantries and via mobile pantries.
Recovery Activities	3.1.3.	Multi-agency resource centers (MARCs) were established throughout affected areas to serve as resource hubs to provide assistance.
		501c3 non-profit long-term recovery groups were established to serve as receiving hubs for donated funds and other resources needed for long-term recovery efforts.
Preparedness Challenges	3.2.1.	General apathy among those meant to be involved in preparedness activities inhibited robust preparedness activities in some locales.
Response Challenges	3.2.2.	Large scope of impact and infrastructure damage from the floods limited access to some areas in need of resources.
		Difficulty managing the large influx of donations of food, water, and other resources that sometimes did not match local needs.
		Language barriers and immigration concerns inhibited some affected households from receiving assistance.
Recovery Challenges	3.2.3.	Difficulty establishing long-term recovery groups and maintaining recovery momentum.
		Stigma, fear, and limited awareness impacted community members’ assistance utilization.
		Administrative barriers inhibited access to federal recovery funds.
Mitigation Recommendations	3.3.1.	Prepare for large-impact, low-frequency disasters.
	3.3.2.	Enhance local-level capacity to plan, respond, and recover.
	3.3.3.	Build inter-organizational relationships and communication channels.
	3.3.4.	Enhance mass-communication capacity among responders and to the public.
	3.3.5.	Leverage technology to coordinate large-scale influx of disjointed donations.
	3.3.6.	Raise public awareness and individual capacity for disaster response through inclusive outreach.
	3.3.7.	Utilize local knowledge to set up shelters and resource sites in ways that promote access and reduce stigma.
	3.3.8.	Food and water needs are situationally dependent (e.g., timing, location, availability of equipment, preferences, etc.). Ensure provisions match need.

## Data Availability

Study data is available upon reasonable request.
